# Editorial: Metabolomics in Infectious Diseases

**DOI:** 10.3389/fgene.2022.875835

**Published:** 2022-03-16

**Authors:** Mahbuba Rahman, Herb Schellhorn, Puthen Veetil Jithesh, Md Mizanur Rahman

**Affiliations:** ^1^ Independent researcher, Toronto, Canada; ^2^ Department of Biology, Faculty of Science, McMaster University, Hamilton, ON, Canada; ^3^ College of Health and Life Sciences, Hamad Bin Khalifa University, Doha, Qatar; ^4^ Biological Science Program, Qatar University, Doha, Qatar

**Keywords:** metabolomics, pandemics, biomarkers, clinical management, therapeutic targets

Metabolomics is an important emerging field of omics technology. While the metabolic pathways in prokaryotic or disease-causing agents are relatively simple compared to those of mammalian hosts, metabolite pools reflect the instant (snapshot) status of cells under healthy or diseased conditions or when infecting the host (Tan et al., 2007) (Lee et al., 2015). In humans, immune cells play a major role in the defense against microbial infection. The metabolic pathways of immune cells are under the stringent control of metabolites and small molecules under a quiescent or active state. Any subtle change in the gene expression during the diseased condition can affect the downstream pathways that consist of proteins and metabolites. Metabolites belong to different chemical groups such as amino acids, organic acid, lipids, or amines. Due to their close association with the cellular system, the detection of metabolites can provide the accurate status of the cell and can also be used as biomarkers of disease or drug targets (Rahman and Hasan 2014). Considering the importance of the field of metabolomics, the current research topic aimed to collect articles where metabolites and metabolite detection tools are used for different purposes in infectious disease. Articles published in this issue show how metabolomics can be used as biomarkers of disease during pandemics, use of metabolomics in clinical management of infectious disease, during metabolomics co-infection of virus and bacteria, metabolites associated with inflammatory disease, and specific proteins associated with transport molecules during communicable and non-communicable disease.

In this issue, an original research article by Taleb et al. (2021) shows that metabolic changes can be used to predict the recovery pattern in critically ill patients caused by the pandemic strain of severe acute respiratory syndrome coronavirus-2 (SARS-CoV-2). Using targeted metabolomics of serum samples, patients that were admitted within 48 h and were under invasive mechanical ventilation (IMV) in the intensive care unit (ICU) had different levels of hypoxanthine and betaine at the first time point of admission. These levels can predict whether the patient will require a short or long stay in the ICU. Another group of metabolites including kynurenine, 3-methylhistidine, ornithine, p-cresol sulfate, C24, and sphingomyelin was measured 1 week later and these could accurately predict the duration of IMV. Another original research conducted by Elrayees et al. (2022) shows that COVID-19 severity is high in patients with type 2 diabetes mellitus and hypertension. Using tandem mass spectrometry as the analytical platform, targeted metabolomics were conducted in serum samples from patients with different disease severity, diabetes status, and hypertension status. Using multivariate and univariate models for data analysis, the authors showed that patients with diabetes and hypertension had increased severity of COVID-19 and reduced levels of specific triacylglycerols. Thus, the studies conducted by Taleb et al. (2021) and Elrayees et al. (2022) clearly show that metabolites, belonging to a class of lipids, have potential for use as novel diagnostic and therapeutic targets and also for clinical management of COVID-19.

Another article that discusses SARS-CoV-2 is by Hasan et al. (2021). This article focuses on detection of the virus in clinical laboratories which is an important consideration of clinical management of the pandemic as this requires rapid detection of the pathogen. The authors review the platforms that are used for detection of SARS-CoV-2. The most frequently used diagnostic methods include the qRT-PCR-based molecular diagnostic method or the metabolomics-based point-of-care (POC) test. Selection of the diagnostic kit used for pathogen detection depends on multiple factors including standardization of sample collection, cost effectiveness, accuracy, and rapid interpretation by clinicians. The authors also review the ongoing development on the metabolomics platform that can be used to detect metabolites from patient samples.

Clinical management of pandemic viruses that are transmitted through respiratory systems also depends on therapeutics that act as antiviral agents. Raihan et al. (2021) review viral outbreaks from the last 2 decades and perform a literature search in PubMed, Scopus, and Google Scholar for potential drug candidates that are metabolites of fungi, bacteria, and microalgae. The authors collected information on 330 metabolites, their source organism, chemical properties, targeted viruses, mechanism of inhibition associated with virus propagation, and IC_50_/EC_50_ values of these metabolites. Although these metabolites have an antiviral effect on a wide range of viruses, their effectiveness against SARS-CoV-2 is unknown. Future research on SARS-COV-2 can provide information on novel antiviral drugs against this pandemic virus.

In addition to viruses, some pathogenic bacteria can spread through the respiratory system and can be life-threatening. Liebenberg et al. (2021) focus on one disease, tuberculosis (TB), which is also considered a global burden and a pandemic disease. The causative agent of TB is *Mycobacterium tuberculosis* and patients suffering from HIV/AIDS (human immunodeficiency virus/acquired immunodeficiency syndrome) are particularly susceptible to TB infection. This particular co-infection is a significant, growing global problem. While immunological deterioration in HIV patients is well known, the effect of this impairment on metabolomics is poorly understood. Through PubMed search, the authors collected information on metabolites that was different in a co-infected patient group compared to an individually infected group of patients. The search results showed that multiple metabolites were found to be differentially regulated in immune cells. The authors concluded that these results are helpful in addressing gaps in existing knowledge on the similarities and deviations in HIV infection and TB infection.

Sakanaka et al. (2021) used biofluid and gas chromatography/mass spectrometry to detect metabolites in saliva and plasma that were associated with an inflammatory disorder, periodontitis. This disease results from an imbalance between the periodontal microbiome and the host response. They found significant differences of the types of metabolites of the lipid metabolic pathway in patients with metabolic syndromes, especially those suffering from cardiovascular (CVD) disease. These findings also reveal that samples from saliva can be used as an alternative to blood tests for detection of periodontitis.

Azad et al. (2021) reviewed the potential of aquaporins, an integral membrane protein found in all living organisms, as a target for infectious disease. Involvement of this membrane protein has been implicated in several non-communicable diseases. The authors emphasized the different modulators of the membrane protein and also provided information on its involvement in different types of microbial pathogenesis and inflammatory responses indicating functional diversity of the protein.

**FIGURE 1:**
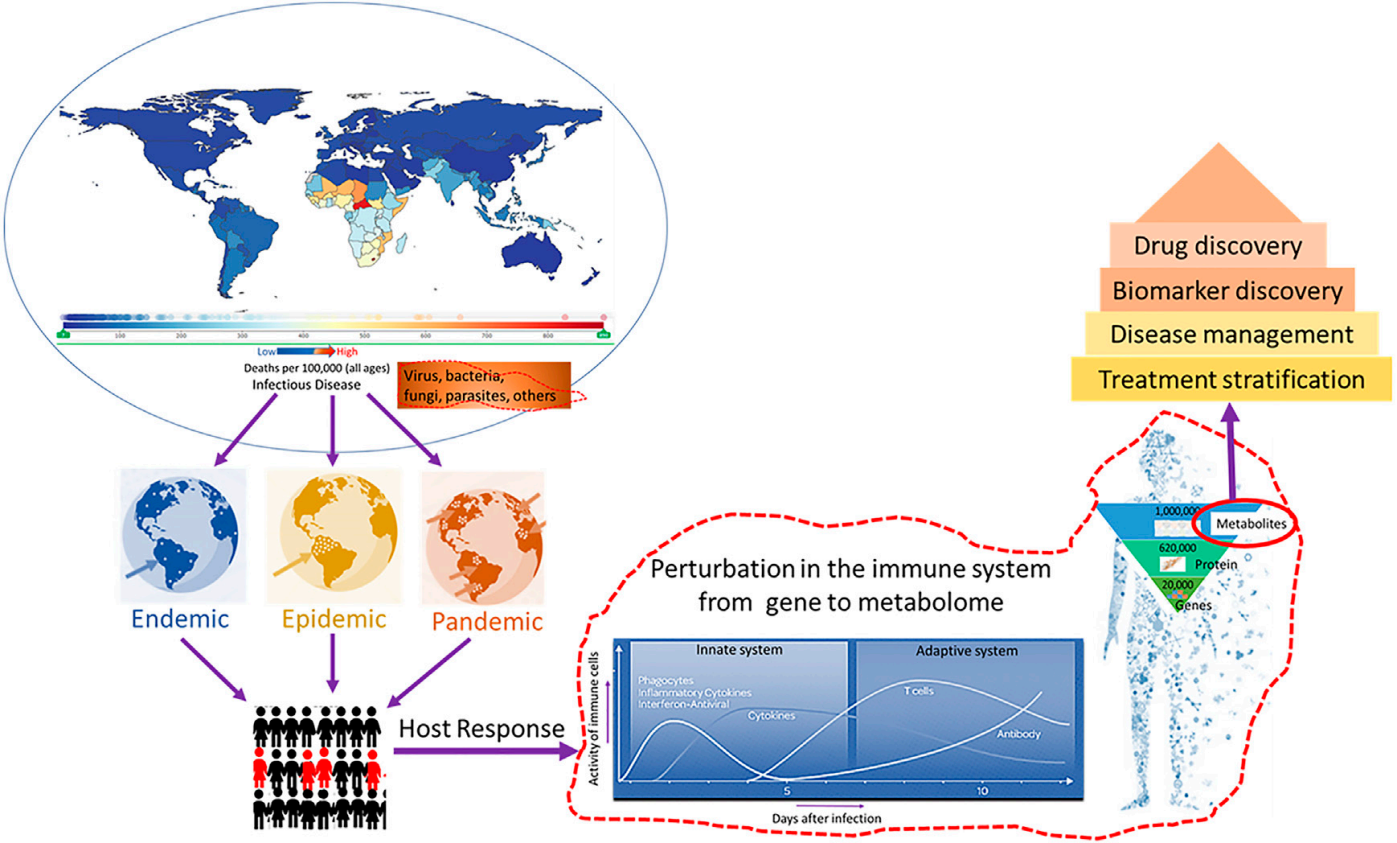
Metabolomics in infectious disease diagnosis, drug discovery, and disease management. Data source: https://vizhub.healthdata.org/gbd-compare/.

In this special issue, researchers showed the potential use of metabolomics for disease diagnosis, prognosis, and drug discovery in infectious disease (Figure 1). We hope that these articles are useful for current and future studies on metabolomics and serve to stimulate new areas of inquiry in infectious disease.
